# [Retracted] MicroRNA-204 inhibits the proliferation, migration and invasion of human lung cancer cells by targeting PCNA-1 and inhibits tumor growth *in vivo*

**DOI:** 10.3892/ijmm.2024.5469

**Published:** 2024-12-09

**Authors:** Ping Li, Qingan Wang, Haining Wang

Int J Mol Med 43: 1149-1156, 2019; DOI: 10.3892/ijmm.2018.4044

Following the publication of this paper, it was drawn to the Editors' attention by a concerned reader that the histological data shown in Fig. 7E were strikingly similar to data appearing in different form in an article written by different authors at different research institutes that had already been published in the journal *International Journal of Molecular Sciences*. In view of the fact that the abovementioned data had already apparently been published prior to its submission to *International Journal of Molecular Medicine*, the Editor has decided that this paper should be retracted from the Journal. The authors were asked for an explanation to account for these concerns, but the Editorial Office did not receive a satisfactory reply. The Editor apologizes to the readership for any inconvenience caused.

